# Digital development and health in China: a serial pathway model through internet use and physical exercise in repeated cross-sectional surveys

**DOI:** 10.3389/fpubh.2026.1976170

**Published:** 2026-09-28

**Authors:** Xiaochu Yang, Minmin Wei, Chenyu Fang, Fengbiao Zhang

**Affiliations:** School of Physical Education, Hunan University of Technology, Zhuzhou, China

**Keywords:** CGSS, China, digital divide, digital exclusion, health equity, health inequality, internet use, physical exercise

## Abstract

**Background:**

Digital exclusion extends beyond access to whether digital participation is associated with health-promoting behavior and health-related outcomes. Physical exercise may form one behavioral link between digital participation and health, but the strength of this sequence may differ across sociodemographic groups.

**Methods:**

We harmonized the 2013, 2018, and 2023 Chinese General Social Survey, including 29,295 adults across 31 provincial-level units. These waves were deliberately selected at approximately 5-year intervals to provide repeated cross-sectional snapshots across a 10-year period. Provincial digital development was measured using a 21-indicator province-year composite index. Survey-weighted population-average path models were supplemented by constrained-model comparisons, formal between-group heterogeneity tests, and multilevel structural equation models separating within- and between-province-year associations. The multilevel models used 87 observed province-year clusters and additionally adjusted for province-year sports-facility resources.

**Results:**

Provincial digital development increased from 0.117 in 2013 to 0.176 in 2018 and was 0.162 in 2023. Internet use increased from 2.132 to 3.593, while physical exercise increased from 2.091 to 2.537. In population-average models, digital development was positively associated with internet use, internet use with physical exercise, and exercise with all three health outcomes; the complete serial indirect associations were statistically supported but small. Multilevel decomposition showed robust within-cluster internet use → exercise → health indirect associations. At the between-cluster level, digital development → internet use and internet use → exercise were supported, whereas exercise → health and the complete digital development → internet use → exercise → health indirect associations were not. Formal heterogeneity tests indicated selective, stage-specific differences by age, hukou, marital status, and education, with weaker evidence for household economic position and no clear evidence for sex.

**Conclusions:**

Digital expansion did not automatically correspond to comparable growth in physical exercise or multidimensional health. The findings support a use-to-benefit perspective while showing that the proposed sequence is level-dependent: individual internet use was consistently linked to exercise and health within province-year contexts, but the complete between-cluster serial association was not supported. Public-health strategies should connect inclusive digital participation with behaviorally specific support and accessible offline opportunities for physical activity.

## Background

Digitalization has changed how people obtain health information, learn about exercise, organize daily life, and maintain social ties. In China, this transformation has been particularly rapid. China had 618 million internet users in 2013 and 1.092 billion in 2023, according to the China Internet Network Information Center. Internet penetration rose from 45.8% to 77.5% during the same period (1, 2). Mobile platforms, short videos, online communities, wearable devices, and location-based applications have substantially expanded residents' opportunities to encounter health and exercise content. However, the expansion of digital access does not necessarily produce corresponding health gains. The internet can reduce the costs of obtaining exercise knowledge, viewing movement demonstrations, finding activity partners, and locating nearby opportunities. However, passive entertainment and sedentary substitution can displace offline activity. Understanding the public-health value of digitalization requires examining whether digital participation becomes sustained health-promoting behavior. It also requires identifying the conditions and populations that shape this conversion. Physical exercise provides an important lens through which to examine this conversion. Regular physical activity reduces the risks of non-communicable disease, functional limitation, premature mortality, and poor mental health (3–6), yet insufficient activity remains widespread worldwide (7–9). Compared with acquiring information, participating in exercise also requires time, self-regulation, social coordination, and suitable activity spaces (10). Physical activity can attenuate some of the health risks associated with prolonged sitting but cannot eliminate them completely (11). The internet is therefore better understood as a potential behavioral resource. It can make exercise practices and opportunities more visible. However, it cannot directly provide safe facilities, affordable services, sufficient time, physical capacity, or durable motivation.

This problem is closely connected to the shift in digital-divide research from whether people can gain access to whether they can obtain benefits. The first-level digital divide concerns devices and network connectivity. The second-level divide shows that even among people who are connected, digital skills, autonomy of use, purposes, and depth of use remain unequal (12–15). The third-level divide moves the question to the tangible outcomes of digital use: who can convert internet participation into valued economic, social, political, and health returns (16–18). Internet diffusion may therefore shift inequality from access to the conversion of benefits rather than eliminate it. This use-to-benefit divide is central to digital inclusion because access and participation are insufficient when their benefits remain limited or unequally distributed. Capability theory similarly distinguishes formally available resources from the actions and outcomes that people are genuinely able to achieve. The internet is likely to support stable offline health practices only when combined with relevant conversion conditions. These conditions include knowledge, time, economic security, social support, physical capacity, and an accessible environment (19). Two competing theoretical expectations follow. Fundamental-cause theory suggests that people with more flexible resources can recognize and use new health opportunities more rapidly, allowing digital participation to reproduce cumulative advantage (20, 21). A resource-compensation perspective offers a different expectation. The internet can lower the costs of professional information, behavioral guidance, and social support. It may therefore provide greater marginal value to people with fewer offline resources (16–18). Whether digital participation widens or narrows disparities in health behavior thus depends on the conditions under which different groups convert digital resources into action. Provincial digital development is not treated as an individual measure of digital exclusion. It represents the contextual digital opportunity environment in which individual digital participation occurs. The third-level digital-divide perspective becomes most relevant at the subsequent use-to-benefit stage, where participation may or may not be associated with behavioral and health-related outcomes.

Existing evidence suggests that the internet can yield health and behavioral benefits, but it does not support a simple claim that technology is inevitably beneficial. Internet-delivered physical activity interventions can produce modest improvements, particularly when information is combined with self-monitoring, goal setting, tailored feedback, and social or professional support (22, 23). Their effects nevertheless vary across target populations, intervention components, and follow-up periods (24–26). Observational studies using large Chinese surveys have generally linked internet use to more frequent exercise or sports participation (27, 28). Internet use has also been associated with better self-rated health, mental health, and multidimensional functioning (29–32). Prospective and longitudinal studies have also reported favorable changes in intrinsic capacity or depressive symptoms (33–35). Contrary evidence also exists. Ji et al. (36) found that greater internet use was associated with less physical activity participation in the 2017 Chinese General Social Survey. Using the 2014 and 2016 China Longitudinal Aging Social Survey, Xie et al. (37) found that internet users were more likely to exhibit depressive tendencies. These divergent findings show that structured digital health interventions differ from general internet use. Interventions combine explicit health goals, behavioral feedback, and professional support. General use also includes work, study, social interaction, shopping, gaming, and passive entertainment. Exercise guidance, social contact, and access to health services may generate benefits, whereas passive entertainment, sleep displacement, and sedentary substitution may produce the opposite pattern (36, 37). Existing research also often uses one survey wave or focuses on older populations. It measures broad internet use without distinguishing purpose. Such research remains vulnerable to selective use by people who are healthier, more active, and better resourced. The same digital content may be actionable for one person but difficult for another to translate into practice. Existing evidence therefore establishes a conditional association that requires further explanation rather than a uniform causal effect. These positive and negative mechanisms can coexist. At the population level, however, the informational, organizational, tracking, and social-support functions of internet use are expected to outweigh sedentary displacement on average, consistent with Chinese survey evidence linking internet use to more frequent exercise or sports participation (27, 28).

Research should therefore move beyond asking whether the internet is beneficial or harmful. Instead, it should identify when, where, and for whom digital participation is converted into exercise and health returns. First, it remains unclear whether the association between internet use and exercise is stable across time. Early internet users tended to have more education, greater economic resources, and stronger technological acceptance. As the internet became everyday infrastructure, user composition and usage patterns changed (12–15). At the same time, exercise videos, online courses, activity booking, route planning, sports communities, and health tracking became more widely available. These changes may have altered the relationship between internet use and offline exercise. A single-year study cannot show whether this association strengthened, weakened, or remained stable during a decade of rapid digitalization. Second, personal internet use does not occur within a uniform regional digital environment. Provincial digital infrastructure, digital industries, applications, human capital, and innovation constitute a broader digital opportunity structure (38). Area-level resources can shape health opportunities independently of individual attributes (39). Provincial digital development may enter individual behavior through several complementary channels. More extensive infrastructure can improve network availability and reliability and reduce the practical costs of routine access. A richer digital-service and application environment can increase the occasions and perceived utility of internet use, while digital-industry development, human capital, and innovation can accelerate service diffusion and normalize digital participation in everyday activities. Provincial digital development is therefore treated as a contextual opportunity structure that may facilitate, but does not determine, individual internet participation. Yet few studies integrate macro digital conditions with individual internet use, exercise, and multiple health outcomes within one staged framework. It therefore remains unclear whether provincial digital development is associated with individual participation and whether that participation connects to exercise and health.

The population boundaries of digital-resource conversion are also unclear. Sex, age, hukou, education, family economic position, and marital status may shape time, digital skills, economic capacity, health needs, and offline exercise opportunities differently. These competing theoretical expectations leave an unresolved question. Existing evidence does not show whether exercise returns concentrate among advantaged groups or compensate people with fewer offline resources. Physical exercise may also constitute an important behavioral link between digital participation and health. Prior studies have separately connected internet use with exercise (27, 28), self-rated health, mental health, and multidimensional functioning (29–32). Longitudinal studies have also reported favorable changes in intrinsic capacity or depressive symptoms (33–35). Few studies, however, have incorporated internet use, exercise, and multiple health outcomes within one analytical framework. The role of exercise in the relationship between digital participation and different health outcomes therefore warrants further examination. The sequential framework and the heterogeneity perspectives therefore serve different analytical roles. The third-level digital-divide and capability perspectives motivate the staged relation among contextual opportunity, digital participation, behavioral conversion, and health-related outcomes. Fundamental-cause theory and the resource-compensation perspective instead provide contrasting interpretations of how particular stages may differ across population groups. Because these expectations may operate differently across stages, subgroup analyses are treated as exploratory assessments of stage-specific heterogeneity rather than as a single confirmatory contest between the two perspectives.

Using harmonized data from the 2013, 2018, and 2023 CGSS waves, this study examines 10-year repeated cross-sectional changes and tests a serial pathway model linking digital development, internet use, physical exercise, and health. First, we examine changes in provincial digital development, internet use, physical exercise, and three health outcomes across the three survey years and assess whether these population-level patterns differ across sociodemographic groups. Second, we examine the sequential associations linking the provincial digital environment with individual digital participation, physical exercise, and health. Based on the preceding theoretical arguments, H1 predicts that higher provincial digital development is associated with more frequent individual internet use. H2 predicts that more frequent internet use is associated with more frequent physical exercise. H3 predicts that more frequent physical exercise is associated with better self-rated health, functional health, and less depressive affect. The serial indirect pathway constitutes the primary theoretical hypothesis: H4 predicts an indirect association between provincial digital development and health through internet use followed by physical exercise. Within this framework, H4a and H4b are secondary competing paths that indicate whether associations remain when one stage of the proposed sequence is bypassed. H4a predicts that internet use remains positively associated with health after accounting for physical exercise, whereas H4b predicts that provincial digital development remains positively associated with physical exercise after accounting for individual internet use. The direct digital-development-to-health paths are estimated without a separate hypothesis. The conceptual framework is shown in Figure 1. Finally, we conduct exploratory subgroup analyses across age, sex, hukou, household economic position, marital status, and education. By treating provincial digital development as contextual digital opportunity, internet use as digital participation, and exercise as behavioral conversion, the framework examines a use-to-benefit dimension of digital inequality without equating area-level digital development with individual digital exclusion.

**Figure 1:**
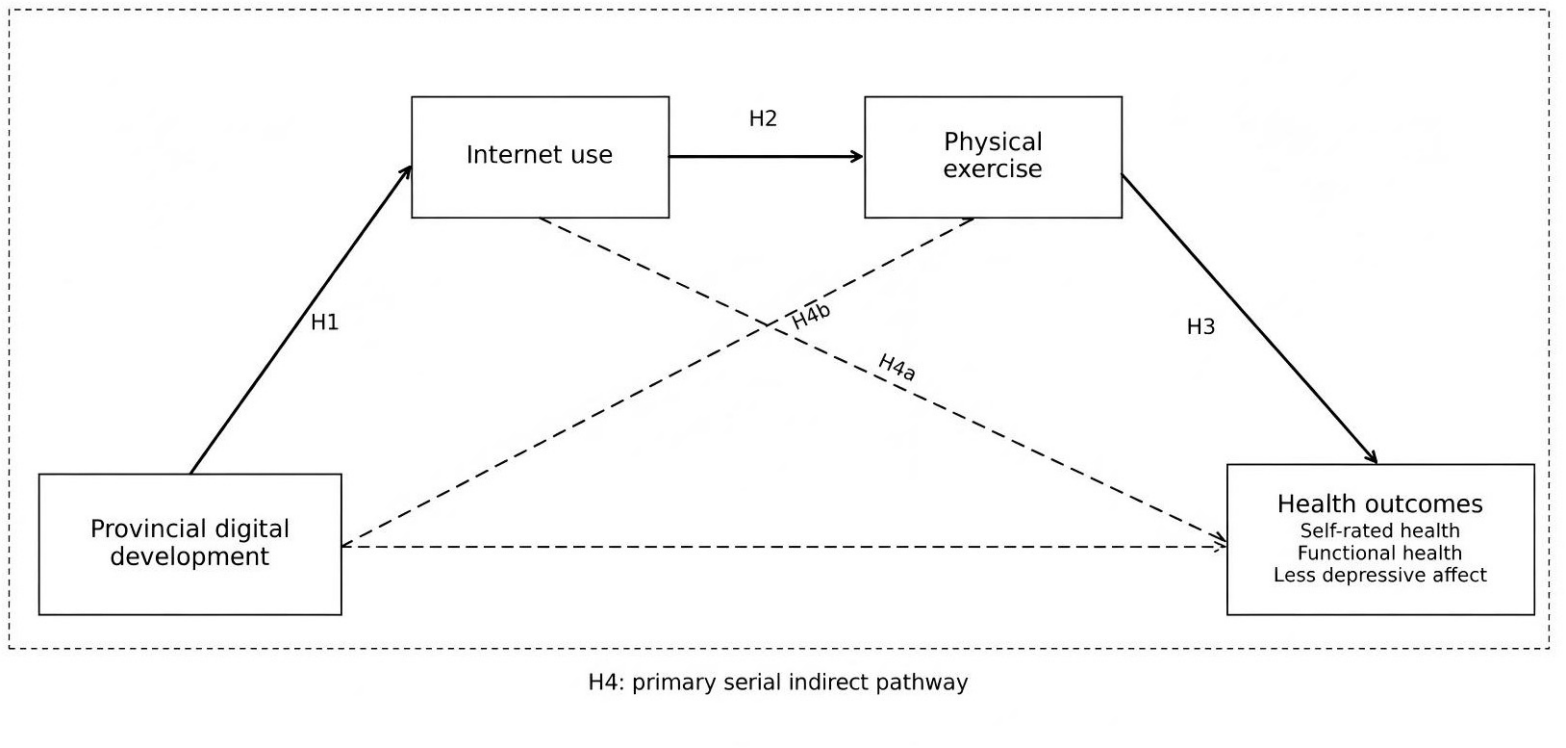
Theoretical model. The model depicts the primary serial indirect pathway linking provincial digital development, internet use, physical exercise, and the three health outcomes. Solid arrows indicate the sequential component paths; dashed arrows indicate secondary competing or additional direct paths. H4 denotes the primary serial indirect hypothesis.

## Methods

### Study design and data sources

The Chinese General Social Survey (CGSS) is a nationally representative repeated cross-sectional survey of adults in mainland China (40). This study integrated the 2013, 2018, and 2023 waves, which initially included 35,551 respondents: 11,438 in 2013, 12,787 in 2018, and 11,326 in 2023. These three waves were deliberately selected at approximately 5-year intervals to provide repeated cross-sectional snapshots across a 10-year period and to compare population-level changes over two comparable 5-year intervals. Respondents were eligible if they were aged 18 years or older and had valid data for all study variables. Required variables included internet use, physical exercise, the 3 health outcomes, covariates, survey weights, and province. Respondents also needed successful matching to the corresponding province-year digital development index. After complete-case analysis, the final analytical sample comprised 29,295 respondents, including 11,266 in 2013, 12,191 in 2018, and 5,838 in 2023. The sample selection process is presented in Figure 2.

**Figure 2:**
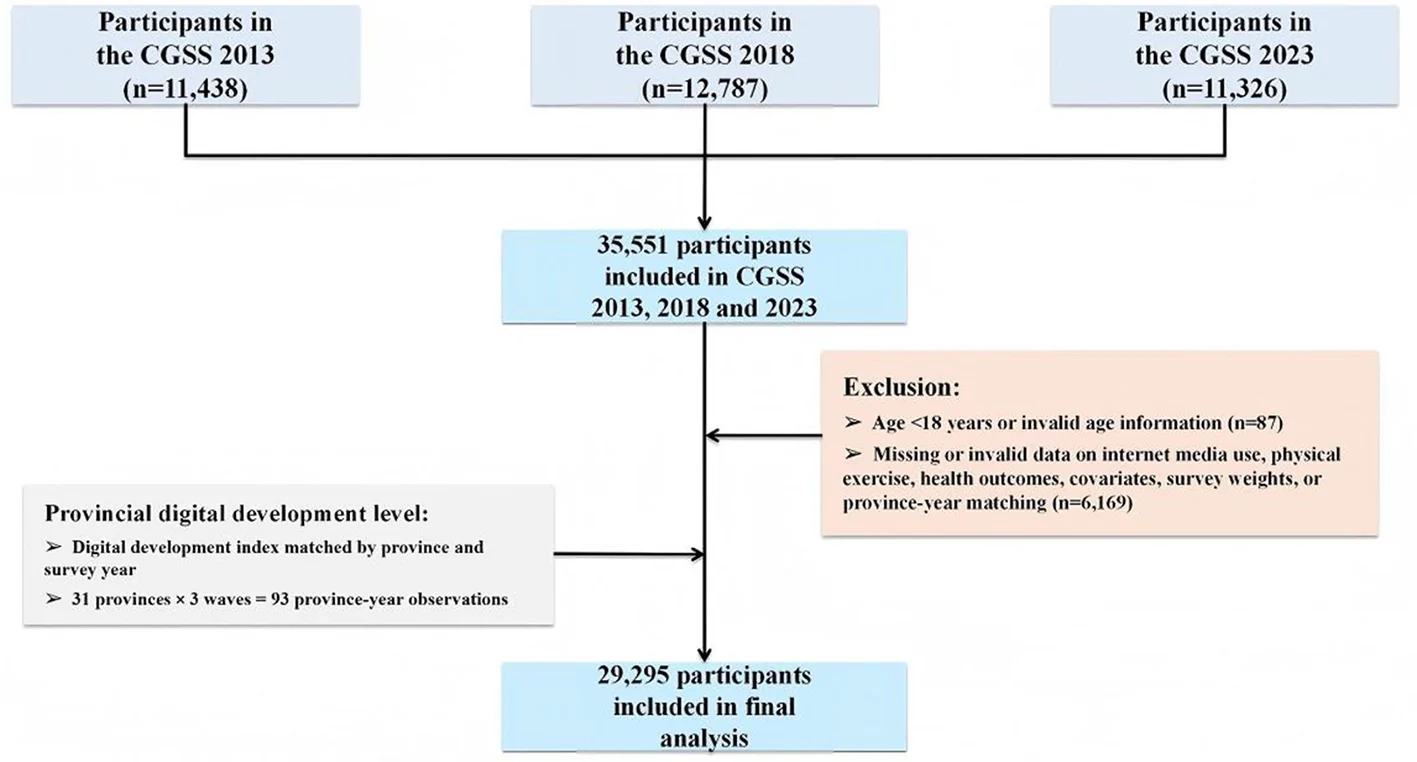
Sample selection for the 2013, 2018, and 2023 CGSS analytic sample. The flowchart shows participant selection within each survey wave, the 93 potential province-year digital-development records, the 87 province-year clusters represented in the final respondent sample, and the final pooled analytic sample.

Province-level digital development data were compiled for 31 provincial-level units in 2013, 2018, and 2023 from official statistical yearbooks and administrative statistical sources. The 21 indicators followed the 3-domain structure proposed for measuring China's digital economy—digital infrastructure, digital-industry development, and the digital-economic environment—and were combined with the entropy-weight method (38). Sources included the China Statistical Yearbook, China Information Industry/Information Yearbook series, and China Tertiary Industry Statistical Yearbook. Additional sources were the China Education Statistical Yearbook and China Science and Technology Statistical Yearbook. Ministry of Industry and Information Technology statistics and CSMAR data were also used. Province-year records were linked to CGSS respondents by 6-digit province code and survey year; all 29,295 analytic records matched.

### Measures

Provincial digital development was the exposure variable. Internet-use frequency was the first mediator, ranging from 1 (never) to 5 (very frequently). Physical-exercise frequency was the second mediator, ranging from 1 (never) to 5 (daily). Three outcomes were examined: self-rated health, functional health, and less depressive affect. All outcomes ranged from 1 to 5, with higher scores indicating better health. The three indicators represented evaluative, functional, and emotional dimensions of health, rather than a single latent health construct. The wording, coding, analytical treatment, and subgroup definitions for all variables are presented in Table 1. Provincial digital development was measured using the framework developed by He et al. (38), with the full 21-indicator system presented in Table 2. All 21 indicators were positively oriented before index construction. Entropy-derived weights were combined with Technique for Order Preference by Similarity to Ideal Solution (TOPSIS) to calculate a province-year composite score. The resulting raw index was matched to respondents by province and survey year and entered as a continuous contextual exposure. The raw index was not standardized before path analysis. Covariates included age, age squared, sex, education, hukou status, marital status, household economic position, and survey year, together with province-year contextual covariates comprising per capita gross domestic product (GDP), urbanization rate, and per capita sports-venue area. Age was grouped into 18–29, 30–44, 45–59, and at least 60 years. Hukou subgroup analyses contrasted rural and urban hukou; 468 other or unclassified cases remained in pooled models but were excluded from this comparison. Household economic position was grouped as lower (categories 1–2) and middle-to-higher (categories 3–5). Marital status was grouped as not currently married and currently married. Education retained the four harmonized categories used in the pooled analysis. Provincial digital development was interpreted as a contextual opportunity measure rather than an individual indicator of digital exclusion.

**Table 1:** Variable definitions and measurements.

Variable	Measurement item or construct	Assignment and analytical treatment
Provincial digital development	Province-year composite based on the 21 indicators in Table 2.	Continuous raw entropy-TOPSIS index (digraw). Matched by province and survey year and entered as the contextual exposure.
Internet use	Frequency of internet/media use during the previous year.	1 = never; 2 = rarely; 3 = sometimes; 4 = often; 5 = very frequently. Entered as the first mediator.
Physical exercise	Frequency of participation in physical exercise.	1 = never; 2 = several times a year or less; 3 = several times a month; 4 = several times a week; 5 = daily.
Self-rated health	Overall assessment of current health.	1 = very poor; 2 = poor; 3 = fair; 4 = good; 5 = very good.
Functional health	Frequency with which health affected work or daily activities.	Reverse-oriented: 1 = always; 2 = often; 3 = sometimes; 4 = rarely; 5 = never.
Less depressive affect	Frequency of feeling depressed or down.	Reverse-oriented: 1 = always; 2 = often; 3 = sometimes; 4 = rarely; 5 = never.
Age	Age in completed years.	Pooled model: age10 = (age – 50)/10 and ageq = age10^2^.
		Subgroup variable agegrp: 1 = 18–29 years (*N* = 3,613); 2 = 30–44 (*N* = 6,877); 3 = 45–59 (*N* = 8,787); 4 = ≥60 (*N* = 10,018).
Sex	Respondent sex.	Original coding: 1 = male; 2 = female. Pooled model: female = 0 for male and 1 for female. Subgroup variable sexgrp: 1 = male (*N* = 14,061); 2 = female (*N* = 15,234).
Education	Highest educational attainment.	Original coding and subgroup variable edugrp: 1 = primary school or below (*N* = 10,467); 2 = junior high (*N* = 8,185); 3 = high school/secondary vocational (*N* = 5,463); 4 = college or above (*N* = 5,180). Pooled model centered these as −1.5, −0.5, 0.5, and 1.5.
Hukou	Household-registration category.	Original coding: 1 = agricultural/rural; 2 = non-agricultural/urban; 3 = other or unclassified. Pooled model used rural as the reference, with separate urban and other/unclassified indicators. Subgroup variable hukgrp: 1 = rural (*N* = 16,434); 2 = urban (*N* = 12,393). Other/unclassified cases (*N* = 468) remained in pooled models but were excluded from rural–urban subgroup analyses.
Marital status	Current marital status.	Pooled model: married = 0 for not currently married and 1 for currently married. Subgroup variable margrp: 1 = not currently married (*N* = 6,728); 2 = currently married (*N* = 22,567).
Household economic position	Subjective family economic position relative to the local average.	Original coding: 1 = far below average; 2 = below average; 3 = average; 4 = above average; 5 = far above average. Pooled model centered these as −2, −1, 0, 1, and 2.
		Subgroup variable econgrp: 1 = lower, original codes 1–2 (*N* = 11,544); 2 = middle-to-higher, original codes 3–5 (*N* = 17,751).
Survey year	CGSS repeated cross-section.	2013, 2018, and 2023. Pooled model: y18 = 1 for 2018 and y23 = 1 for 2023; 2013 was the reference year.
Survey weight	Wave-specific CGSS main survey weight.	Restricted to positive valid values and normalized to a mean of 1 within each survey wave. The same normalized survey weight was used in pooled and subgroup analyses.
Per capita GDP	Per capita GDP	Continuous province-year variable, expressed in yuan per person.
Urbanization rate	Proportion of the urban population in the regional population.	Continuous province-year variable, calculated as urban population divided by total regional population.
Per capita sports-venue area	Regional sports-venue area per capita.	Continuous province-year variable, expressed as sports-venue area per person.

X, provincial digital development; M1, internet use; M2, physical exercise; Y1, self-rated health; Y2, functional health; Y3, less depressive affect.

**Table 2:** Provincial digital development measurement system.

First-level domain	Second-level domain	Third-level indicator	Unit	Primary data source	Weight
Digital infrastructure	Hardware	Long-haul fiber-optic cable length	10,000 km	China Statistical Yearbook	0.0540
		Internet broadband access ports	10,000	China Statistical Yearbook	0.0507
		Mobile telephone base stations	10,000	China Statistical Yearbook	0.0498
	Software	Internet domain names	10,000	China Statistical Yearbook	0.0434
		IPv4 addresses	10,000	China Statistical Yearbook	0.0386
		Internet websites	10,000	China Statistical Yearbook; MIIT	0.0458
Digital-industry development	Digital industrialization	Software business revenue	CNY 100 million	China Statistical Yearbook; information-industry yearbook before 2013	0.0385
		Total telecommunications business	CNY 100 million	China Statistical Yearbook	0.0467
		Electronic-information manufacturing enterprises	Number	CSMAR; above-designated-size enterprises	0.0393
	Industrial digitalization	Websites per 100 enterprises	Number	China Statistical Yearbook	0.0559
		Enterprises conducting e-commerce transactions	%	China Statistical Yearbook	0.0528
		E-commerce sales	CNY 100 million	China Statistical Yearbook	0.0425
		Computers per 100 persons	Number	China Statistical Yearbook	0.0538
Digital-economic environment	Application	Mobile internet users	10,000 users	China Statistical Yearbook	0.0520
		Mobile telephone users	10,000 users	China Statistical Yearbook	0.0527
		Digital television users	10,000 households	China Tertiary Industry Statistical Yearbook	0.0524
	Human capital	Information-sector employment share	%	China Tertiary Industry Statistical Yearbook	0.0493
		Bachelor's graduates	Persons	China Education Statistical Yearbook	0.0532
	Innovation	R&D personnel full-time equivalent	Person-years	China Science and Technology Statistical Yearbook	0.0475
		R&D institutions	Number	China Science and Technology Statistical Yearbook	0.0380
		Patents granted	Number	China Statistical Yearbook	0.0432

All indicators are positively oriented. Weights were estimated from the province-year indicator panel using the entropy-weight method and sum to 1.0000 apart from rounding.

MIIT, Ministry of Industry and Information Technology; CSMAR, China Stock Market & Accounting Research database.

### Statistical analysis

All analyses incorporated CGSS survey weights, normalized to a mean of 1 within each survey wave (41, 42). Descriptive statistics were first used to summarize demographic characteristics and the major study variables across the 2013, 2018, and 2023 samples. Survey-weighted means were calculated for provincial digital development, internet use, physical exercise, and the three health outcomes, and survey-weighted regressions were used to examine repeated cross-sectional trends across survey years and sociodemographic groups. Correlation analyses and model diagnostics were then conducted. Harman's single-factor test assessed whether the self-reported individual-level variables were dominated by a single common factor (43, 44), variance inflation factors were used to assess multicollinearity (45), and Pearson correlations summarized bivariate associations.

Population-average serial path models were subsequently estimated using normalized survey weights, robust maximum likelihood estimation, and province-year cluster-robust standard errors (54). Provincial digital development was specified as the exposure, internet use as the first intermediate variable, physical exercise as the second intermediate variable, and self-rated health, functional health, and less depressive affect as separate outcomes. Models adjusted for age, age squared, sex, education, hukou status, marital status, household economic position, survey year, per capita GDP, urbanization rate, and per capita sports-venue area. Direct, specific indirect, serial indirect, total indirect, and total associations were estimated. To further assess the contribution of the sequential component paths, constrained multilevel models were estimated by fixing the between-level internet use → physical exercise path, the physical exercise → health path, and both paths jointly to zero.

Exploratory subgroup analyses were conducted by age, sex, hukou status, household economic position, marital status, and education. In addition to subgroup-specific pathway estimates, formal between-group heterogeneity was assessed using interaction terms, Wald tests, and contrasts of the complete serial indirect association.

Finally, multilevel structural equation models (MSEM) were estimated to separate the within- and between-province-year components of the 2–1–1 structure, in which provincial digital development was measured at the province-year level and internet use, physical exercise, and health outcomes were measured at the individual level (46). Respondents were nested within province-year clusters (52). Provincial digital development, per capita GDP, urbanization rate, per capita sports-venue area, and survey-year indicators were specified at the between level, whereas internet use, physical exercise, and the health outcomes were decomposed into within- and between-cluster components. All MSEM models used normalized survey weights and MLR estimation in Mplus version 7.4 (Muthén & Muthén, Los Angeles, CA, United States). IBM SPSS Statistics version 26.0 (IBM Corp., Armonk, NY, United States) was used for descriptive statistics, correlations, diagnostics, and trend analyses. All tests were two-sided, with *P* < 0.05 indicating statistical significance (53).

### Use of generative AI in manuscript preparation

During manuscript preparation, the authors used OpenAI ChatGPT (GPT-5.6 Thinking; OpenAI) for English-language translation and document formatting. The authors reviewed, verified, and edited all outputs and take full responsibility for the analyses and text.

## Results

### Common method bias and multicollinearity

Most individual-level variables were self-reported. Harman's unrotated factor analysis of the 11 individual-level survey variables extracted four factors with eigenvalues >1. The first factor explained 30.40% of the total variance. This result indicates that the observed covariance was not dominated by a single common factor, although it does not rule out common method bias. In addition, the provincial digital-development index was derived from external statistical data and therefore did not share the same response method as the individual-level variables. VIFs ranged from 1.003 to 2.396, and tolerance values ranged from 0.417 to 0.997, indicating no serious multicollinearity among the substantive predictors.

### Descriptive statistics and correlations

The final analytic sample included 29,295 respondents, comprising 11,266 in 2013, 12,191 in 2018, and 5,838 in 2023. Table 3 presents survey-weighted trends in the six key variables across the three survey years. Overall, the provincial digital development index increased from 0.117 in 2013 to 0.176 in 2018 and remained at 0.162 in 2023. The linear trend was statistically significant (*P* for trend < 0.001). Internet use increased from 2.132 in 2013 to 3.660 in 2018 and remained relatively high at 3.593 in 2023. Physical exercise increased more modestly, with weighted means of 2.091, 2.493, and 2.537 across the three survey years. Both internet use and physical exercise showed significant positive linear trends (both *P* for trend < 0.001). However, the three health indicators did not improve in parallel with digital participation. Survey-weighted means for self-rated health were 3.627, 3.914, and 3.353 in 2013, 2018, and 2023, respectively. Functional health means were 3.895, 4.229, and 3.711, while less depressive affect means were 3.881, 3.940, and 3.804. All three health indicators reached relatively higher levels in 2018, but their 2023 means were lower than those observed in 2013. Self-rated health and functional health showed significant negative linear trends (both *P* for trend < 0.001). Less depressive affect also showed a negative linear trend (*P* for trend = 0.001). Figure 3 further presents ten-year repeated-cross-sectional patterns of the six key variables across 16 sociodemographic subgroups. Overall, provincial digital development and internet use increased across all subgroups. Physical exercise also increased across groups, but its growth was generally smaller than that of internet use. Internet use remained relatively lower among older adults, rural-hukou residents, economically disadvantaged individuals, and those with lower educational attainment. Health patterns showed greater heterogeneity across subgroups. Several groups showed no clear linear changes in functional health or less depressive affect. Table 4 reports survey-weighted Pearson correlations among the six key variables. Provincial digital development was positively correlated with internet use (*r* = 0.226) and physical exercise (*r* = 0.139). Internet use was positively correlated with physical exercise (*r* = 0.249), self-rated health (*r* = 0.306), functional health (*r* = 0.308), and less depressive affect (*r* = 0.134). Physical exercise was also positively correlated with self-rated health (*r* = 0.137), functional health (*r* = 0.161), and less depressive affect (*r* = 0.111). All reported correlations were statistically significant (all *P* < 0.001). Given the large analytic sample, statistical significance was interpreted together with coefficient magnitude; several bivariate correlations were modest despite *P* < 0.001.

**Table 3:** Overall survey-weighted trends in the six key variables, 2013–2023.

Variable	2013	2018	2023	*P* for trend
Provincial digital development	0.117	0.176	0.162	< 0.001
Internet use	2.132	3.660	3.593	< 0.001
Physical exercise	2.091	2.493	2.537	< 0.001
Self-rated health	3.627	3.914	3.353	< 0.001
Functional health	3.895	4.229	3.711	< 0.001
Less depressive affect	3.881	3.940	3.804	0.001

Values are survey-weighted means. *P* for trend was obtained from survey-weighted linear regression across the three survey years.

**Figure 3:**
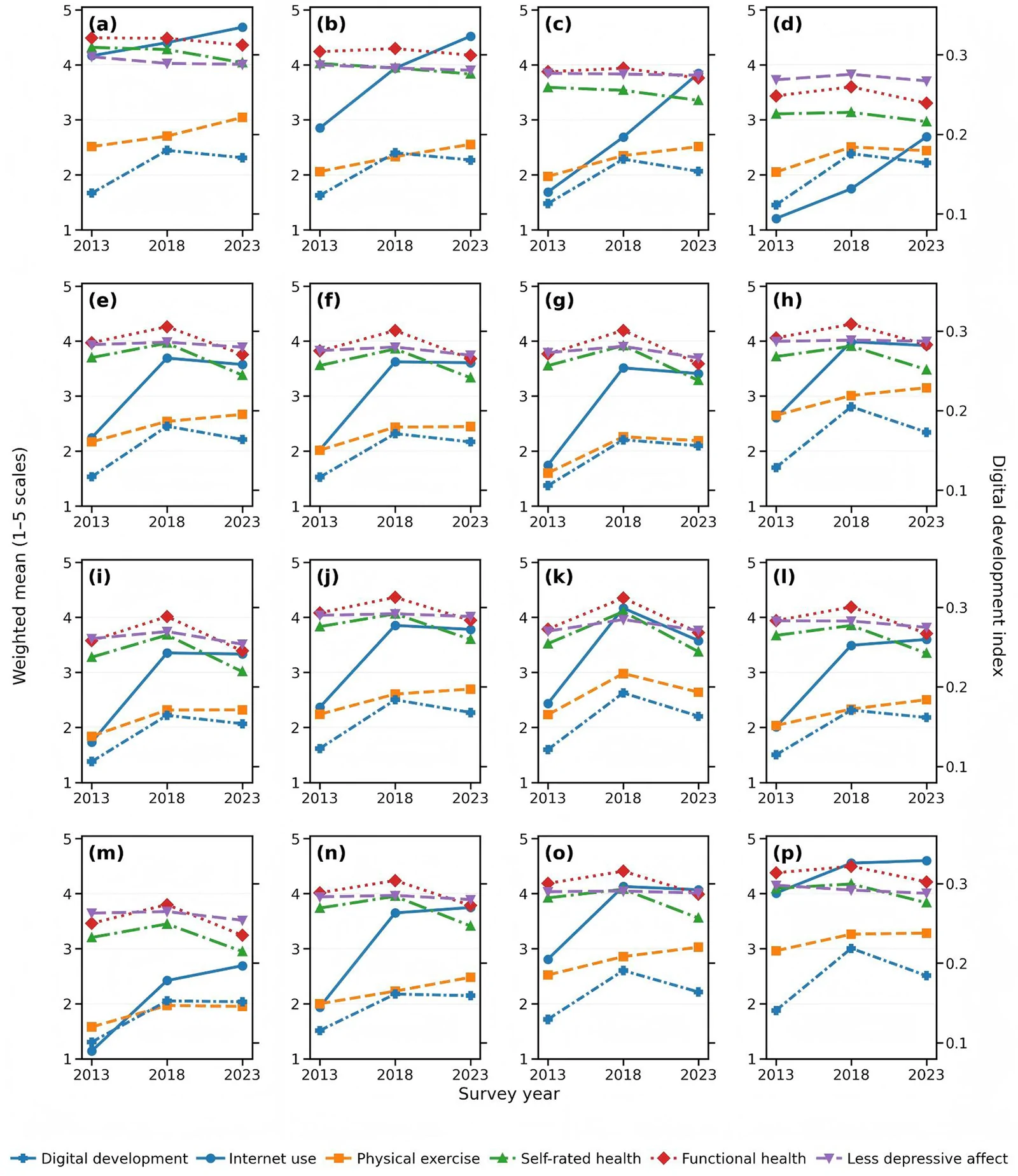
Ten-year repeated cross-sectional patterns in the six key variables across sociodemographic subgroups. Lines show survey-weighted survey-wave means. The left axis applies to internet use, physical exercise, self-rated health, functional health, and less depressive affect. The right axis applies to provincial digital development. **(a–h)** represent age 18–29, age 30–44, age 45–59, age 60+, male, female, rural hukou, and urban hukou. **(i–p)** represent lower and middle-to-higher economic position, not currently married, currently married, primary or below, junior high, high school/vocational, and college or above.

**Table 4:** Survey-weighted descriptive statistics and Pearson correlations.

Variable	*M*	*SD*	1	2	3	4	5	6
1. Provincial digital development	0.150	0.105	—					
2. Internet use	3.059	1.679	0.226^***^	—				
3. Physical exercise	2.347	1.508	0.139^***^	0.249^***^	—			
4. Self-rated health	3.692	1.092	0.086^***^	0.306^***^	0.137^***^	—		
5. Functional health	3.997	1.095	0.103^***^	0.308^***^	0.161^***^	0.652^***^	—	
6. Less depressive affect	3.890	0.983	0.044^***^	0.134^***^	0.111^***^	0.406^***^	0.493^***^	—

M and SD are survey-weighted. Pearson correlations are survey-weighted.

^***^P < 0.001.

### Relationship between provincial digital development and health: a serial pathway test

The population-average cluster-robust serial path results are presented in Tables 5, 6. Provincial digital development was positively associated with internet use [*B* = 0.734, *SE* = 0.258, *P* = 0.004, 95% CI (0.230, 1.239)], supporting H1. Internet use was positively associated with physical exercise [*B* = 0.082, *SE* = 0.016, *P* < 0.001, 95% CI (0.052, 0.113)], supporting H2. Physical exercise was positively associated with self-rated health (*B* = 0.046, *P* < 0.001), functional health (*B* = 0.054, *P* < 0.001), and less depressive affect (*B* = 0.034, *P* < 0.001), supporting H3. The primary serial hypothesis was then evaluated. The complete population-average serial indirect associations were 0.003 for self-rated health (*P* = 0.011), 0.003 for functional health (*P* = 0.012), and 0.002 for less depressive affect (*P* = 0.020), with 95% confidence intervals excluding or reaching zero only by rounding. These coefficients were small and represented approximately 1.0%, 0.8%, and 4.7% of the respective total associations. By comparison, the specific indirect associations through internet use alone were 0.041, 0.048, and 0.019, indicating that the exercise-containing serial component was substantially smaller than the internet-use-only component. Within the competing paths specified around H4, internet use remained positively associated with all three health outcomes after physical exercise was included (supporting H4a), whereas the direct provincial digital-development-to-exercise association was not statistically supported [*B* = 0.473, *SE* = 0.264, *P* = 0.073, 95% CI (−0.045, 0.992); H4b not supported]. These population-average estimates are interpreted as statistical associations rather than causal mediation.

**Table 5:** Component path coefficients from the cluster-robust serial path models.

Outcome	Predictor	R^2^	*B*	Robust *SE*	*z*	*P*	95% CI
Internet use	Provincial digital development	0.568	0.734	0.258	2.851	0.004	[0.230, 1.239]
Physical exercise	Internet use	0.169	0.082	0.016	5.265	< 0.001	[0.052, 0.113]
	Provincial digital development		0.473	0.264	1.791	0.073	[−0.045, 0.992]
Self-rated health	Physical exercise	0.232	0.046	0.007	6.593	< 0.001	[0.032, 0.060]
	Internet use		0.056	0.010	5.670	< 0.001	[0.036, 0.075]
	Provincial digital development		0.222	0.218	1.017	0.309	[−0.206, 0.649]
Functional health	Physical exercise	0.201	0.054	0.008	7.043	< 0.001	[0.039, 0.068]
	Internet use		0.065	0.008	8.467	< 0.001	[0.050, 0.080]
	Provincial digital development		0.285	0.212	1.341	0.180	[−0.132, 0.701]
Less depressive affect	Physical exercise	0.073	0.034	0.008	4.292	< 0.001	[0.018, 0.049]
	Internet use		0.025	0.008	3.351	0.001	[0.010, 0.040]
	Provincial digital development		0.006	0.326	0.019	0.985	[−0.633, 0.645]

Unstandardized coefficients are reported. Robust SEs and 95% CIs account for clustering within 87 province-year units. Models used normalized survey weights and MLR estimation. All equations adjusted for age, age squared, sex, education, hukou status, marital status, household economic position, and survey year. *N* = 29,295.

**Table 6:** Indirect and total associations in the serial path models.

Association	Estimate	*SE*	*z*	*P*	95% CI
Digital development and physical exercise					
Digital development → internet use → physical exercise	0.060	0.024	2.536	0.011	[0.014, 0.107]
Total: digital development → physical exercise	0.534	0.269	1.984	0.047	[0.006, 1.061]
Self-rated health					
Digital development → internet use → self-rated health	0.041	0.016	2.546	0.011	[0.009, 0.072]
Digital development → physical exercise → self-rated health	0.022	0.012	1.769	0.077	[−0.002, 0.046]
Digital development → internet use → physical exercise → self-rated health	0.003	0.001	2.527	0.011	[0.001, 0.005]
Total indirect: digital development → self-rated health	0.065	0.021	3.072	0.002	[0.024, 0.107]
Total: digital development → self-rated health	0.287	0.217	1.322	0.186	[−0.138, 0.712]
Internet use → physical exercise → self-rated health	0.004	0.001	3.894	< 0.001	[0.002, 0.006]
Total: internet use → self-rated health	0.059	0.010	6.142	< 0.001	[0.040, 0.078]
Functional health					
Digital development → internet use → functional health	0.048	0.017	2.783	0.005	[0.014, 0.081]
Digital development → physical exercise → functional health	0.025	0.015	1.725	0.085	[−0.003, 0.054]
Digital development → internet use → physical exercise → functional health	0.003	0.001	2.502	0.012	[0.001, 0.006]
Total indirect: digital development → functional health	0.076	0.025	3.112	0.002	[0.028, 0.124]
Total: digital development → functional health	0.361	0.213	1.697	0.090	[−0.056, 0.778]
Internet use → physical exercise → functional health	0.004	0.001	4.373	< 0.001	[0.002, 0.006]
Total: internet use → functional health	0.069	0.008	8.921	< 0.001	[0.054, 0.085]
Less depressive affect					
Digital development → internet use → less depressive affect	0.019	0.008	2.321	0.020	[0.003, 0.034]
Digital development → physical exercise → less depressive affect	0.016	0.010	1.576	0.115	[−0.004, 0.036]
Digital development → internet use → physical exercise → less depressive affect	0.002	0.001	2.333	0.020	[0.000, 0.004]
Total indirect: digital development → less depressive affect	0.037	0.013	2.849	0.004	[0.011, 0.062]
Total: digital development → less depressive affect	0.043	0.326	0.132	0.895	[−0.596, 0.682]
Internet use → physical exercise → less depressive affect	0.003	0.001	3.203	0.001	[0.001, 0.004]
Total: internet use → less depressive affect	0.028	0.007	3.781	< 0.001	[0.014, 0.043]

Indirect, total indirect, and total associations were calculated in Mplus 7.4 using MODEL CONSTRAINT. SEs and 95% CIs are delta-method estimates; bootstrap resampling was not used. All models used *N* = 29,295, 87 province-year clusters, normalized survey weights, MLR estimation.

Constrained-model comparisons provided additional evidence on the component structure of the sequence (Table 7). Fixing the between-level internet use → physical exercise path to zero significantly worsened model fit for self-rated health (χ^2^ = 15.907, df = 1, *P* < 0.001), functional health (χ^2^ = 15.668, df = 1, *P* < 0.001), and less depressive affect (χ^2^ = 15.531, df = 1, *P* < 0.001). In contrast, fixing the between-level physical exercise → health path to zero did not significantly worsen fit for any outcome (*P* = 0.375, 0.508, and.727, respectively). Jointly fixing both paths worsened fit for all three outcomes (all *P* < 0.001), a result primarily attributable to the internet use → physical exercise restriction. These comparisons support the internet-to-exercise component but do not provide model-fit evidence for a complete between-cluster serial chain through exercise.

**Table 7:** Constrained multilevel model comparisons for the sequential component paths.

Health outcome	Between-level restriction	χ^2^	df	*P*	Interpretation
Self-rated health	Internet use → exercise = 0	15.907	1	< 0.001	Fit worsened
	Exercise → health = 0	0.788	1	0.375	No significant worsening
	Both paths = 0	21.236	2	< 0.001	Fit worsened
Functional health	Internet use → exercise = 0	15.668	1	< 0.001	Fit worsened
	Exercise → health = 0	0.439	1	0.508	No significant worsening
	Both paths = 0	22.820	2	< 0.001	Fit worsened
Less depressive affect	Internet use → exercise = 0	15.531	1	< 0.001	Fit worsened
	Exercise → health = 0	0.122	1	0.727	No significant worsening
	Both paths = 0	23.360	2	< 0.001	Fit worsened

Restricted MSEM models used N = 29,295, 87 province-year clusters, normalized survey weights, MLR estimation. The unrestricted model was saturated; reported robust model-fit chi-square values therefore test the imposed restriction relative to the unrestricted model.

### Subgroup analysis of the serial pathway

Figures 4, 5 decompose subgroup-specific serial associations into component pathways and complete serial indirect associations, identifying where the digital-to-health pathway weakened across groups. Age-stratified results showed a relatively clear gradient. Among adults aged 18–29, digital development–internet use was 0.389 [95% CI (−0.105, 0.884)]. Internet use–exercise was −0.008 [95% CI (−0.138, 0.121)], and both confidence intervals included zero. Exercise associations with all three health outcomes also included zero, and none of the three serial indirect associations received statistical support. Among adults aged 30–44, internet use–exercise was 0.126 [95% CI (0.090, 0.163)]. Exercise associations with self-rated health (SRH) and functional health (FH) were also supported. However, digital development–internet use was 0.589 [95% CI (−0.067, 1.246)], with its confidence interval including zero. Accordingly, all three serial indirect associations included or reached zero. Among adults aged 45–59, digital development–internet use was 1.035 [95% CI (0.297, 1.773)]. Internet use–exercise was 0.121 [95% CI (0.086, 0.157)], and exercise was positively associated with all three health outcomes. The serial association for FH was 0.007 [95% CI (0.001, 0.014)], while the SRH and less depressive affect (LDA) intervals had lower bounds near zero. Among adults aged 60 or older, digital development–internet use was 1.148 [95% CI (0.477, 1.819)]. Internet use–exercise was 0.103 [95% CI (0.072, 0.135)], and exercise associations with all three health outcomes excluded zero. Serial associations were 0.009 for SRH, 0.013 for FH, and 0.007 for LDA. Their respective 95% CIs were [0.003, 0.016], [0.004, 0.022], and [0.002, 0.012].

**Figure 4:**
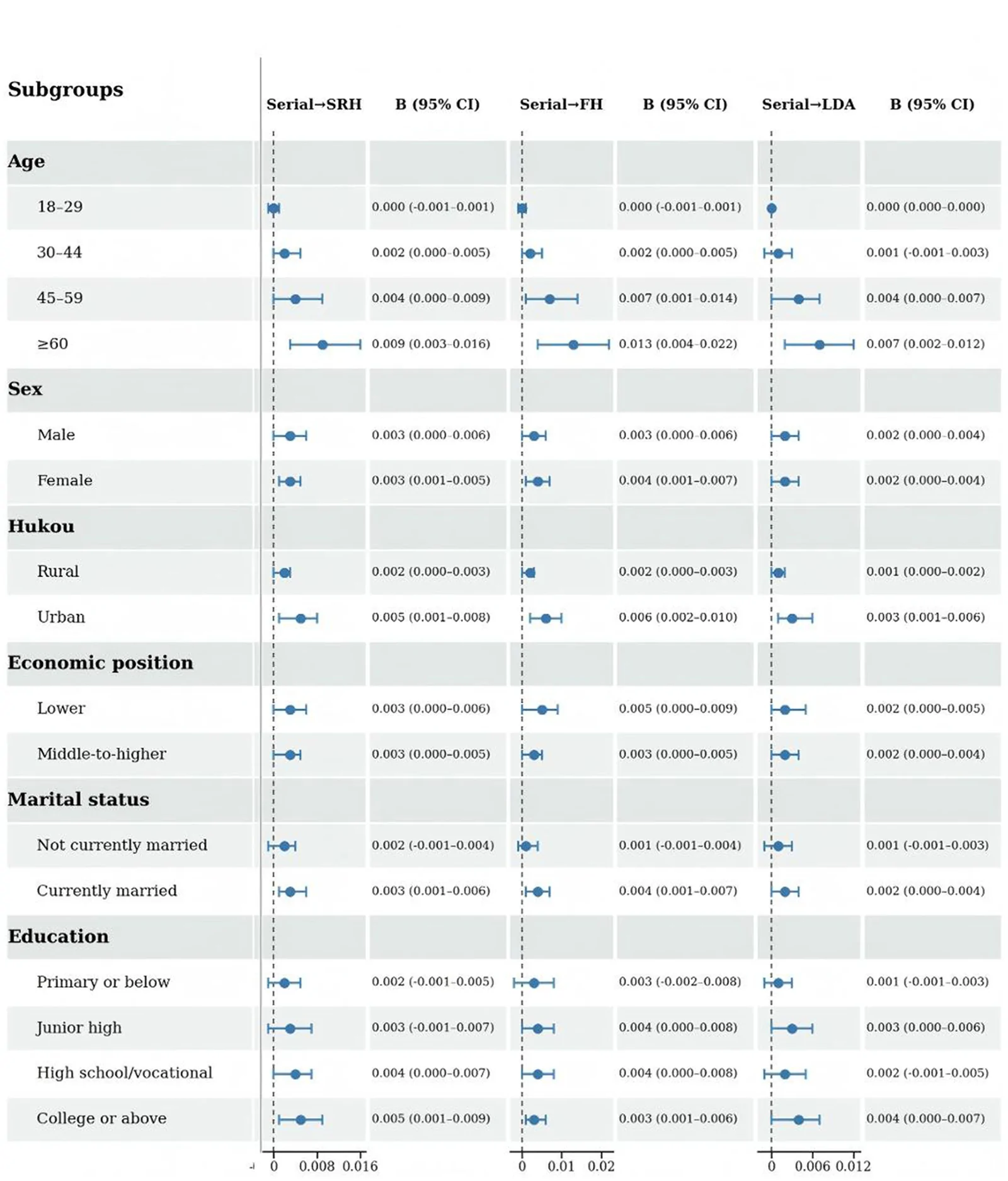
Component pathways across sociodemographic subgroups. Forest plots show unstandardized coefficients **(***B***)** with 95% confidence intervals from subgroup-specific cluster-robust serial path models. The five panels show digital development → internet use, internet use → exercise, and exercise associations with SRH, FH, and LDA. Each pathway uses its own horizontal scale, with the dashed vertical line indicating *B* = 0. Models incorporated normalized survey weights and province-year cluster-robust standard errors. SRH indicates self-rated health; FH, functional health; LDA, less depressive affect. These subgroup-specific estimates are descriptive; formal interaction and Wald tests are reported in Table 8.

**Figure 5:**
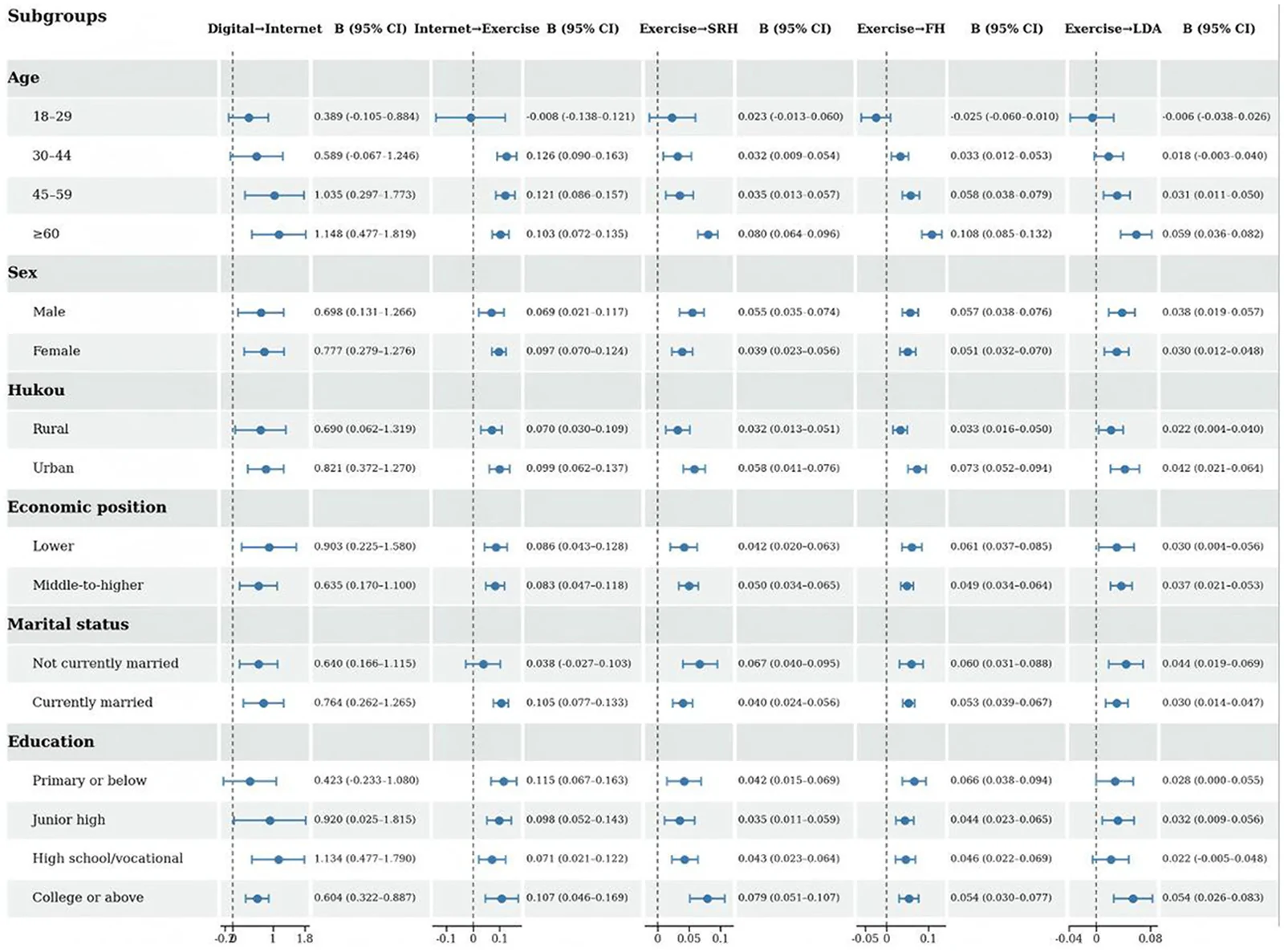
Serial indirect associations across sociodemographic subgroups. Forest plots show unstandardized complete serial indirect associations **(***B***)** with 95% confidence intervals across sociodemographic subgroups. The serial pathway is provincial digital development → internet use → physical exercise → health. Separate panels present SRH, FH, and LDA. Each panel uses its own horizontal scale, with the dashed vertical line indicating *B* = 0. Models incorporated normalized survey weights and province-year cluster-robust standard errors. Formal between-group interaction, Wald, and serial-indirect contrast tests are reported in Table 8. SRH indicates self-rated health; FH, functional health; LDA, less depressive affect.

Sex and hukou analyses showed that pathway attenuation was not always attributable to a single unsupported component path. Among males, digital development–internet use was 0.698 [95% CI (0.131, 1.266)]. Internet use–exercise was 0.069 [95% CI (0.021, 0.117)], and exercise–health associations also had confidence intervals excluding zero. However, the lower bounds of all three serial indirect associations reached zero, indicating attenuation across the complete sequence. Among females, the first two pathway coefficients were 0.777 [95% CI (0.279, 1.276)] and 0.097 [95% CI (0.070, 0.124)]. Serial associations were 0.003 for SRH [95% CI (0.001, 0.005)] and 0.004 for FH [95% CI (0.001, 0.007)]. The lower bound for the LDA serial association was close to zero. Among rural-hukou respondents, component pathways generally excluded zero, whereas all three serial associations had lower bounds at or near zero. Among urban-hukou respondents, serial associations were 0.005 for SRH and 0.006 for FH. Their 95% CIs were [0.001, 0.008] and [0.002, 0.010], respectively. The LDA serial association was 0.003 [95% CI (0.001, 0.006)], and all three confidence intervals excluded zero.

Household economic position and marital status revealed additional patterns of pathway attenuation. In the lower economic group, digital development–internet use was 0.903 [95% CI (0.225, 1.580)]. Internet use–exercise was 0.086 [95% CI (0.043, 0.128)], but lower bounds for the three serial associations reached or approached zero. In the middle-to-higher economic group, major component pathways remained positive, although lower bounds for the serial associations were also close to zero. Marital status revealed a clearer behavioral-conversion breakpoint. Among respondents not currently married, digital development–internet use was 0.640 [95% CI (0.166, 1.115)]. However, internet use–exercise was only 0.038 [95% CI (−0.027, 0.103)], with its confidence interval crossing zero. The confidence intervals for all three serial indirect associations also crossed zero. Among currently married respondents, internet use–exercise was 0.105 [95% CI (0.077, 0.133)], and the three serial associations showed a more stable pattern.

Education showed another stage-specific pattern. In the primary-or-below group, internet use–exercise was 0.115 [95% CI (0.067, 0.163)], and exercise was positively associated with all three health outcomes. However, digital development–internet use was 0.423 [95% CI (−0.233, 1.080)], with its confidence interval crossing zero. This pattern suggests attenuation when the macro digital environment translated into individual digital participation. In the junior-high group, digital development–internet use was 0.920 [95% CI (0.025, 1.815)]. Internet use–exercise was 0.098 [95% CI (0.052, 0.143)], and component pathways were generally stable. However, lower bounds for all three serial associations reached or approached zero. In the high-school or vocational group, digital development–internet use was 1.134 [95% CI (0.477, 1.790)]. Internet use–exercise was 0.071 [95% CI (0.021, 0.122)], but exercise–LDA was 0.022 [95% CI (−0.005, 0.048)]. This pattern indicates attenuation at the health-return stage for LDA. In the college-or-above group, digital development–internet use was 0.604 [95% CI (0.322, 0.887)]. Internet use–exercise was 0.107 [95% CI (0.046, 0.169)], and exercise associations with all three health outcomes excluded zero. Serial associations were 0.005 for SRH [95% CI (0.001, 0.009)] and 0.003 for FH [95% CI (0.001, 0.006)]. The lower bound for the LDA serial association was close to zero.

Overall, the descriptive subgroup estimates in Figures 4, 5 suggested attenuation at different stages, but formal tests showed that heterogeneity was selective rather than universal. Joint Wald tests supported heterogeneity across age for all three outcomes (all *P* < 0.001), hukou (*P* = 0.001–0.003), marital status (all *P* < 0.001), and education (*P* = 0.002–0.033). Evidence for household economic position was weaker and outcome-specific, while no joint test reached *P* < 0.05 for sex. Most formal differences occurred in component paths rather than in the complete serial indirect association. The clearest serial contrasts occurred for adults aged 45–59 relative to those aged 18–29: *B* = 0.008 for self-rated health (*P* = 0.008), 0.013 for functional health (*P* = 0.002), and 0.007 for less depressive affect (*P* = 0.007). Hukou heterogeneity was concentrated mainly in the internet use → exercise path, and marital-status differences reflected opposing changes in the digital development → internet use and internet use → exercise stages. Education showed component-path heterogeneity without statistically supported differences in the complete serial indirect association. Table 8 reports the formal tests in full. The subgroup analyses remain exploratory because no universal cumulative-advantage or resource-compensation pattern was prespecified.

**Table 8:** Formal between-group heterogeneity tests.

Dimension	SRH omnibus *P*	FH omnibus *P*	LDA omnibus *P*	Notable component-path contrasts, Δ*B* (*P*)	Complete serial indirect contrast, Δ*B* (*P*)
Age	< 0.001	< 0.001	< 0.001	45–59 vs. 18–29: Digital development → Internet use, 1.744 (0.001); Exercise → FH, 0.086 (< 0.001); Exercise → LDA, 0.037 (0.020)	45–59 vs. 18–29: SRH, 0.008 (0.008); FH, 0.013 (0.002); LDA, 0.007 (0.007)
Sex	0.138	0.147	0.097	No statistically significant component-path contrast	No statistically significant contrast
Hukou	0.001	< 0.001	0.003	Urban vs. Rural: Internet use → Physical exercise, −0.086 (< 0.001)	SRH, −0.002 (0.123); FH, −0.002 (0.239); LDA, −0.001 (0.163)
Economic position	0.083	0.038	0.097	Middle-to-higher vs. Lower: Internet use → Physical exercise, −0.030 (0.079)	SRH, −0.003 (0.066); FH, −0.005 (0.050); LDA, −0.002 (0.214)
Marital status	< 0.001	< 0.001	< 0.001	Currently married vs. Not currently married: Digital development → Internet use, 1.025 (0.001); Internet use → Physical exercise, −0.086 (< 0.001)	SRH, 0.002 (0.350); FH, 0.004 (0.148); LDA, 0.002 (0.339)
Education	0.002	0.009	0.033	Junior high vs. Primary or below: Internet use → Physical exercise, −0.071 (0.006)	No statistically significant contrast across the three outcomes

ΔB coefficients are interaction contrasts relative to the stated reference group. Omnibus P values are from joint Wald tests of between-group heterogeneity in the digital development → internet use, internet use → physical exercise, and physical exercise → health paths. Complete serial indirect contrasts were estimated using MODEL CONSTRAINT.

SRH, self-rated health; FH, functional health; LDA, less depressive affect.

### Multilevel structural equation modeling analysis

The multilevel structural equation models separated within- and between-province-year associations for the three health outcomes (Table 9). All models converged normally with *N* = 29,295 respondents nested in 87 province-year clusters. At the within level, internet use was positively associated with physical exercise in all three models (*B* ≈ 0.081, *P* < 0.001). Physical exercise was positively associated with self-rated health (*B* = 0.054, *P* < 0.001), functional health (*B* = 0.054, *P* < 0.001), and less depressive affect (*B* = 0.036, *P* < 0.001). The within-level internet use → physical exercise → health indirect associations were 0.004 for self-rated health, 0.004 for functional health, and 0.003 for less depressive affect (all *P* < 0.001).

**Table 9:** Multilevel structural equation model results for within- and between-province-year associations.

Level	Association	SRH B [95% CI], *P*	FH B [95% CI], *P*	LDA B [95% CI], *P*
Within	Internet use → physical exercise	0.081 [0.056, 0.106], < 0.001	0.081 [0.056, 0.106], < 0.001	0.081 [0.056, 0.106], < 0.001
	Physical exercise → health	0.054 [0.042, 0.066], < 0.001	0.054 [0.042, 0.066], < 0.001	0.036 [0.024, 0.048], < 0.001
	Internet use → health	0.051 [0.037, 0.065], < 0.001	0.050 [0.036, 0.064], < 0.001	0.016 [0.002, 0.030], 0.017
	Internet use → physical exercise → health	0.004 [0.002, 0.006], < 0.001	0.004 [0.002, 0.006], < 0.001	0.003 [0.001, 0.005], < 0.001
Between	Digital development → internet use	1.861 [1.173, 2.549], < 0.001	1.831 [1.147, 2.515], < 0.001	1.844 [1.166, 2.522], < 0.001
	Internet use → physical exercise	0.802 [0.608, 0.996], < 0.001	0.797 [0.597, 0.997], < 0.001	0.801 [0.601, 1.001], < 0.001
	Physical exercise → health	−0.099 [−0.303, 0.105],0.342	0.054 [−0.101, 0.209], 0.498	0.031 [−0.149, 0.211],0.732
	Internet use → health	0.321 [0.100, 0.542],0.004	0.323 [0.180, 0.466], < 0.001	0.255 [0.063, 0.447],0.009
	Digital development → internet use → health	0.598 [0.153, 1.043],0.009	0.591 [0.262, 0.920], < 0.001	0.470 [0.060, 0.880],0.025
	Digital development → internet use → physical exercise → health	−0.147 [−0.463, 0.169],0.362	0.078 [−0.145, 0.301],0.493	0.046 [−0.215, 0.307],0.728

Unstandardized coefficients are reported. The MSEM included N = 29,295 respondents nested within 87 province-year clusters and used normalized survey weights and MLR estimation. Provincial digital development, per capita GDP, urbanization rate, per capita sports-venue area, and survey-year indicators were specified at the between level. Individual-level covariates included age, sex, education, hukou status, marital status, and household economic position. SRH, self-rated health; FH, functional health; LDA, less depressive affect.

At the between level, provincial digital development was strongly associated with average internet use for self-rated health (*B* = 1.861, *P* < 0.001), functional health (*B* = 1.831, *P* < 0.001), and less depressive affect (*B* = 1.844, *P* < 0.001). Average internet use was also associated with average physical exercise (*B* = 0.797–0.802, all *P* < 0.001). However, the between-level physical exercise → health path was not statistically supported for self-rated health (*B* = −0.099, *P* = 0.342), functional health (*B* = 0.054, *P* = 0.498), or less depressive affect (*B* = 0.031, *P* = 0.732). Correspondingly, the complete between-level serial indirect association was not statistically supported for self-rated health (*B* = −0.147, *P* = 0.362), functional health (*B* = 0.078, *P* = 0.493), or less depressive affect (*B* = 0.046, *P* = 0.728). In contrast, the digital development → internet use → health indirect association remained supported for all three outcomes. The multilevel evidence therefore indicates a level-dependent pattern rather than a fully supported between-cluster serial chain.

## Discussion

### Principal findings

This study examined repeated cross-sectional changes and a staged association linking provincial digital development, individual internet use, physical exercise, and multidimensional health across three national survey waves. Internet use increased much more than physical exercise, while the three health indicators did not improve in parallel. The population-average models showed positive digital development → internet use, internet use → exercise, and exercise → health associations and small complete serial indirect associations. However, the MSEM clarified an important level boundary. Within province-year contexts, internet use was consistently associated with exercise and, through exercise, with all three health outcomes. At the between-cluster level, digital development was strongly associated with internet use and internet use with exercise, but average exercise was not associated with average health after the other between-level paths were considered. Accordingly, the complete between-level serial indirect association was not statistically supported. Constrained-model comparisons reached the same conclusion: the between-level internet use → exercise path contributed to model fit, whereas constraining exercise → health to zero did not significantly worsen fit. Formal subgroup tests further showed that heterogeneity was selective and stage-specific rather than universal. These results represent statistical associations from repeated cross-sectional data rather than causal mediation.

### Digital opportunity, digital exclusion, health equity, and the use-to-benefit divide

Our findings speak to the third-level digital-divide literature by examining how digital participation is associated with behavioral and health-related outcomes within different contextual and sociodemographic conditions (16–18). This perspective is directly relevant to health equity because digital inclusion cannot be evaluated by connectivity alone. The decade-long trends illustrate this distinction. Internet use rose sharply, but exercise increased more modestly and health did not move in the same direction. Digital diffusion may therefore leave a use-to-benefit divide even when access and participation become more widespread. This interpretation complements Li et al. (47), who showed that internet access remained consequential for healthy aging among older Chinese adults. Their study emphasized connectivity, whereas our results locate an additional potential inequality after access and participation. The provincial index itself should not be read as an individual digital-exclusion measure; it represents the broader digital opportunity context within which unequal participation and use-to-benefit patterns may emerge.

Provincial digital development was associated with more frequent internet use, but its direct association with exercise was not statistically supported. By contrast, the indirect association from digital development through internet use to exercise was supported. This pattern suggests that favorable digital environments may create opportunities for participation without automatically producing offline health behavior. That distinction is consistent with the capability approach, which separates available resources from the actions and outcomes people can actually achieve (19). Digital infrastructure and services may expand opportunities, but behavioral returns still depend on personal, social, and offline conversion conditions.

The results also show that digital participation linked health through more than one pathway. Indirect associations through internet use alone were supported across all three health outcomes, whereas the digital-development-to-exercise-specific pathway was not supported. Thus, exercise represents one conversion route within a broader set of potential digital health returns.

### Physical exercise as a behavioral conversion pathway

The positive internet use-exercise association supports previous Chinese survey evidence linking digital participation with more frequent exercise or sports participation (27, 28). However, the magnitude of the association was modest, which is plausible because general internet use is not a structured physical-activity intervention. General use includes work, entertainment, shopping, social interaction, and passive browsing alongside exercise and health content (15, 36). Effective digital activity interventions usually add goal setting, self-monitoring, tailored feedback, and social or professional support (22, 23, 25). Our general frequency measure captures none of these behavior-change components.

The internet may therefore increase exposure to exercise knowledge and opportunities without guaranteeing sustained activity. Time, physical capacity, motivation, facilities, and social support remain necessary for translating information into behavior (10). The exercise-to-health paths were positive across all three outcomes in the population-average models and at the within-cluster level. However, the corresponding between-cluster exercise-to-health paths were not statistically supported. Physical exercise therefore appears to be a plausible individual-level behavioral link without establishing a complete province-year-level serial mechanism.

The population-average complete serial coefficients were small, and the internet-use-only indirect associations were substantially larger than the exercise-containing serial components. Internet use also retained associations with all three health outcomes after exercise was included. Exercise should therefore be interpreted as one modest behavioral pathway rather than the dominant explanation for internet-related health differences. This conclusion complements Liao et al. (48), who identified self-efficacy, health literacy, and exercise within a broader chain-mediation framework. It also accords with Chen et al. (34), who found social participation to be an important pathway between internet use and intrinsic capacity. Future mechanism research should therefore consider several behavioral, informational, social, and health-service pathways simultaneously.

### Sociodemographic differentiation in the serial pathway

The subgroup results add depth by showing that sociodemographic heterogeneity occurred at selected stages rather than across the entire sequence uniformly. Formal Wald tests supported overall heterogeneity by age, hukou, marital status, and education across the three health models. Age produced the clearest differences in the complete serial association: adults aged 45–59 differed significantly from those aged 18–29 for self-rated health, functional health, and less depressive affect. The ≥60 group showed larger descriptive serial coefficients than the youngest group, but the formal serial contrasts were not statistically significant.

This age pattern is compatible with evidence that digital engagement can be particularly relevant to health and functioning in later life (34, 47, 49, 50). Older adults may have more salient health needs, making health-oriented digital information and exercise guidance more actionable. These explanations remain interpretive even though formal between-group tests were conducted, because the subgroup analysis was exploratory and the repeated cross-sectional design does not identify the mechanisms producing the differences.

Education and marital status revealed stage-specific heterogeneity. Education showed a significant omnibus pattern across all three outcomes, with the clearest component difference occurring in the internet use → exercise path for junior-high respondents relative to the primary-or-below group; however, none of the education-group contrasts in the complete serial indirect association was statistically significant. Marital status also showed significant omnibus heterogeneity. Currently married respondents had a stronger digital development → internet use association but a weaker internet use → exercise association than those not currently married, producing no significant difference in the complete serial indirect association.

Hukou heterogeneity was concentrated mainly at the participation-to-exercise stage: the internet use → exercise association was weaker for urban than rural hukou in the formal interaction model, while the complete serial indirect contrasts were not statistically significant. Household economic position provided only limited, outcome-specific evidence of heterogeneity, and sex showed no statistically supported omnibus differences. Taken together, the formal tests do not support one universal cumulative-advantage or resource-compensation gradient. Instead, the findings are more consistent with multiple stage-specific configurations of opportunity, participation, and behavioral constraints.

### Public health implications for digital inclusion and health equity

These findings support a public-health shift from digital access alone toward the equitable conversion of digital participation into feasible health behavior. Connectivity and usable interfaces remain necessary, particularly where digital participation remains limited (47, 51). However, health equity also depends on whether people can translate digital opportunities into sustained physical exercise. Digital-health and health-promotion programs should therefore provide behaviorally specific support, including attainable goals, self-monitoring, tailored feedback, social encouragement, and links to affordable local exercise opportunities (22, 23, 25).

The subgroup findings further suggest that interventions should consider the stage at which conversion constraints appear, while avoiding assumptions that every descriptive subgroup pattern reflects a confirmed difference. Formal tests indicated the clearest heterogeneity for age, hukou, marital status, and education. For groups with weaker internet use → exercise associations, digital resources may need stronger links to feasible activity opportunities, social support, and local services. The absence of consistent sex or economic-position differences in the full sequence also cautions against universal demographic targeting. These implications remain exploratory and should be tested prospectively.

Evaluation should also move beyond penetration and platform engagement. Programs should assess whether digital resources increase meaningful use, physical exercise, and multidimensional health outcomes. Digital development should therefore complement, rather than replace, safe spaces, affordable facilities, and trained physical-activity personnel.

### Strengths, limitations, and future research

This study has several strengths. It integrates three large national survey waves spanning a decade and links respondents with a transparent 21-indicator province-year digital development measure. It combines survey weights with population-average cluster-robust estimates, constrained-model comparisons, formal heterogeneity tests, and MSEM that explicitly separates within- and between-province-year associations. It also examines three complementary health outcomes and adds province-year sports-facility resources as a contextual control. The staged framework distinguishes the macro digital environment, individual participation, behavioral conversion, and health-related outcomes while allowing their associations to differ by analytical level.

Several limitations require caution. First, repeated cross-sectional data cannot establish temporal ordering, within-person change, or causal mediation. Reverse causation and residual confounding therefore remain plausible throughout the proposed sequence. Second, internet use, exercise, and health were measured with broad ordinal self-reports. Internet frequency does not distinguish health-information seeking, exercise content, social communication, work, or passive entertainment, and exercise frequency does not capture duration, intensity, or activity type. Third, the provincial digital index is spatially coarse and cannot represent neighborhood facilities, walkability, or local service accessibility. Although the multilevel models additionally controlled province-year sports-facility resources, unmeasured contextual factors such as economic development, urbanization, and healthcare resources may still confound between-cluster associations. The 87 observed province-year clusters also limit the precision of more highly parameterized between-level models.

Fourth, the three survey waves were intentionally selected at approximately five-year intervals to represent broad decade-scale population snapshots. This design does not capture shorter-term fluctuations or non-linear changes occurring between 2013, 2018, and 2023. Fifth, complete-case analysis and cross-wave harmonization may affect representativeness and measurement precision. Finally, subgroup analyses remained exploratory despite the addition of formal interaction and serial-contrast tests, and multiple comparisons warrant cautious interpretation. Future studies should use panel designs, richer digital-use measures, objective activity indicators, finer geographic linkage, and a larger number of repeated contextual units to evaluate temporal ordering and stage-specific multilevel mechanisms.

## Conclusions

Between 2013 and 2023, internet use expanded much faster than physical exercise, while multidimensional health did not improve in parallel. Provincial digital development was consistently associated with greater internet use, and internet use was associated with more frequent physical exercise. Within province-year contexts, internet use was also indirectly associated with all three health outcomes through physical exercise. However, the complete between-level serial association from provincial digital development through internet use and exercise was not statistically supported, indicating that contextual digital opportunity does not automatically correspond to average health differences through exercise. Formal subgroup tests further showed selective, stage-specific heterogeneity rather than a universal social gradient. These findings frame digital inequality as a use-to-benefit problem while emphasizing that behavioral and health-related associations depend on both analytical level and social context. Public-health strategies should pair inclusive digital participation with behaviorally specific support and accessible offline opportunities for physical activity.

## Data Availability

The data analyzed in this study is subject to the following licenses/restrictions: The dataset has several limitations. First, the CGSS is a repeated cross-sectional survey, so the same individuals are not followed over time and within-person changes cannot be assessed. Second, internet use, physical exercise, and health outcomes are based on self-reported measures and may be subject to reporting bias. The harmonized measures are relatively broad and do not capture internet-use purposes or the duration and intensity of physical exercise. Third, only three survey waves (2013, 2018, and 2023) were included, limiting the assessment of non-linear temporal changes. Fourth, the digital-development index was measured at the provincial level and cannot capture finer local differences in digital environments. Finally, the analysis used complete cases, which may introduce some selection bias. Participant-level CGSS data are also subject to third-party access and registration requirements and cannot be redistributed by the authors. Requests to access these datasets should be directed to Chinese General Social Survey (CGSS), https://cgss.ruc.edu.cn/English/Home.htm.
